# Targeting the Type 5 Metabotropic Glutamate Receptor: A Potential Therapeutic Strategy for Neurodegenerative Diseases?

**DOI:** 10.3389/fphar.2022.893422

**Published:** 2022-05-11

**Authors:** Rebecca F. Budgett, Geor Bakker, Eugenia Sergeev, Kirstie A. Bennett, Sophie J. Bradley

**Affiliations:** ^1^ The Centre for Translational Pharmacology, Institute of Molecular, Cell and Systems Biology, College of Medical, Veterinary and Life Sciences, University of Glasgow, Glasgow, United Kingdom; ^2^ Sosei Heptares, Cambridge, United Kingdom

**Keywords:** GPCR, Alzheimer’s disease, neurodegenerative disease, neuroinflammation, G protein coupled receptors, drug discovery

## Abstract

The type 5 metabotropic glutamate receptor, mGlu_5_, has been proposed as a potential therapeutic target for the treatment of several neurodegenerative diseases. In preclinical neurodegenerative disease models, novel allosteric modulators have been shown to improve cognitive performance and reduce disease-related pathology. A common pathological hallmark of neurodegenerative diseases is a chronic neuroinflammatory response, involving glial cells such as astrocytes and microglia. Since mGlu_5_ is expressed in astrocytes, targeting this receptor could provide a potential mechanism by which neuroinflammatory processes in neurodegenerative disease may be modulated. This review will discuss current evidence that highlights the potential of mGlu_5_ allosteric modulators to treat neurodegenerative diseases, including Alzheimer’s disease, Huntington’s disease, Parkinson’s disease, and amyotrophic lateral sclerosis. Furthermore, this review will explore the role of mGlu_5_ in neuroinflammatory responses, and the potential for this G protein-coupled receptor to modulate neuroinflammation.

## Introduction

Neurodegenerative diseases are characterized by the progressive degeneration of neurons in the central nervous system (CNS). Common neurodegenerative diseases include Alzheimer’s disease, Huntington’s disease, amyotrophic lateral sclerosis, and Parkinson’s disease. These diseases give rise to behavioral changes and cognitive decline (Karantzoulis and Galvin, 2011). In 2019, there were over 55 million people living with dementia worldwide and it is predicted that this will increase to 83 million by 2030, and to 153 million by 2050 (Dementia Forecasting Collaborators, 2022). Most current treatments are symptomatic rather than disease-modifying in nature, and the large, predicted increase in individuals living with dementia globally means that there is an urgent need for novel therapeutic interventions.

The G protein-coupled receptor (GPCR) superfamily represents the largest group of transmembrane receptors in the human genome and are the most common target for clinically approved drugs (Santos et al., 2016; Sriram and Insel, 2018). Metabotropic glutamate receptors (mGlu), which mediate slow, long-lasting responses to the endogenous ligand, glutamate, (Conn and Pin, 1997), are members of this superfamily and can be divided into eight subtypes split further into three groups based on factors such as their sequence homology and pharmacological profile. The type 5 metabotropic glutamate receptor (mGlu_5_) belongs to group 1 mGlu receptors, along with the mGlu_1_ receptor. Although both mGlu_1_ and mGlu_5_ have been implicated in neurodegenerative processes, this review focusses on mGlu_5_ as a potential target for therapeutic intervention in neurodegenerative disorders.

## The Type 5 Metabotropic Glutamate Receptor

### Distribution

Within the brain, mGlu_5_ is expressed in the cerebral cortex, olfactory blub, hippocampus, striatum, and basal ganglia with highest expression rates in the hippocampus and basal ganglia where they modulate reward processing and movement (Shigemoto et al., 1993; Wong et al., 2013). It is also expressed in the spinal cord (Valerio et al., 1997). It is predominately localised on the postsynaptic membrane of glutamatergic neurons, although it can also be found on the presynaptic membrane (Luján et al., 1996) where they are suggested to play a role in autoregulation of glutamatergic exocytosis modulating the extracellular level of glutamate available to bind to postsynaptic glutamatergic receptors (Pittaluga, 2016). Postsynaptically, around 50%–80% of mGlu_5_ is expressed on intracellular membranes such as the nuclear membranes and endoplasmic reticulum (Hubert, Paquet and Smith, 2001; Kumar, Jong and O’Malley, 2008), where its activation results in a sustained response from extracellular signal-regulated protein kinase (ERK1/2) stimulated phosphorylation of the transcription factor Elk-1 (Jong, Kumar and O’Malley, 2009) which mediates long-term depression (LTD) (Purgert et al., 2014). Activation of mGlu_5_ on the plasma membrane results in a rapid calcium response (Jong, Kumar and O’Malley, 2009) which leads to both LTD and long-term potentiation (LTP) (Purgert et al., 2014). This differential expression impacts upon pharmacology, with ligands needing to either diffuse or be transported across membranes in order to reach intracellular receptors (Jong et al., 2005).

In addition to expression in neurons, mGlu_5_ is expressed in glial cells, including astrocytes and microglia, where its activation is important for glial cell function and their interaction with neurons (Loane et al., 2012). Astrocytes, star-like cells with processes that extend from their cell body, are the most prevalent glial cell type in the brain, accounting for between 20% and 50% of total CNS number (Hasel and Liddelow, 2021). They play many roles in the brain, including providing trophic support to neurons, mediating synapse formation, and regulating neurotransmitter uptake (Hasel and Liddelow, 2021). In astrocytes isolated from rodent tissue, mGlu_5_ is found in cells isolated from the hippocampus, cortex, thalamus, tegmentum and striatum, but not in the cerebellum or spinal cord (Biber et al., 1999; Silva et al., 1999). In addition, the expression of mGlu_5_ in astrocytes is highest during neurodevelopment in both cells isolated from rodent tissue (postnatal day 7) and in rodent brain slices (postnatal days 1–10) and then decreases with age (Cai et al., 2000), which suggests that its role may change as the brain develops. Preclinical models have identified an upregulation of astrocytic mGlu_5_ in several diseases including Alzheimer’s disease (Lim et al., 2013; Shrivastava et al., 2013), amyotrophic lateral sclerosis (Anneser et al., 2004; Vermeiren et al., 2006), and multiple sclerosis (Fulmer et al., 2014). Analysis of post-mortem tissue from patients have corroborated these findings, showing upregulation of astrocytic mGlu_5_ in and around lesions (Aronica et al., 2001; Anneser et al., 2004; Newcombe et al., 2008; Casley et al., 2009; Lim et al., 2013).

Microglia, which at rest have many ramified processes extending from their small cell body, are mediators of the brain’s innate immune response. They play a key role in maintaining homeostasis in the brain by responding to extracellular signals in their microenvironment and clearing away any toxic substances of cellular debris (Hanisch and Kettenmann, 2007). Similar to astrocytic cultures, mGlu_5_ is expressed in microglia isolated from rodent tissue, but at lower levels than in astrocytes (Byrnes et al., 2009). This mGlu_5_ expression is up-regulated in the activated microglia that surround lesions following spinal cord or brain injury in rodents (Byrnes et al., 2009; Drouin-Ouellet et al., 2011).

### Pharmacological Modulation

**FIGURE 1:**
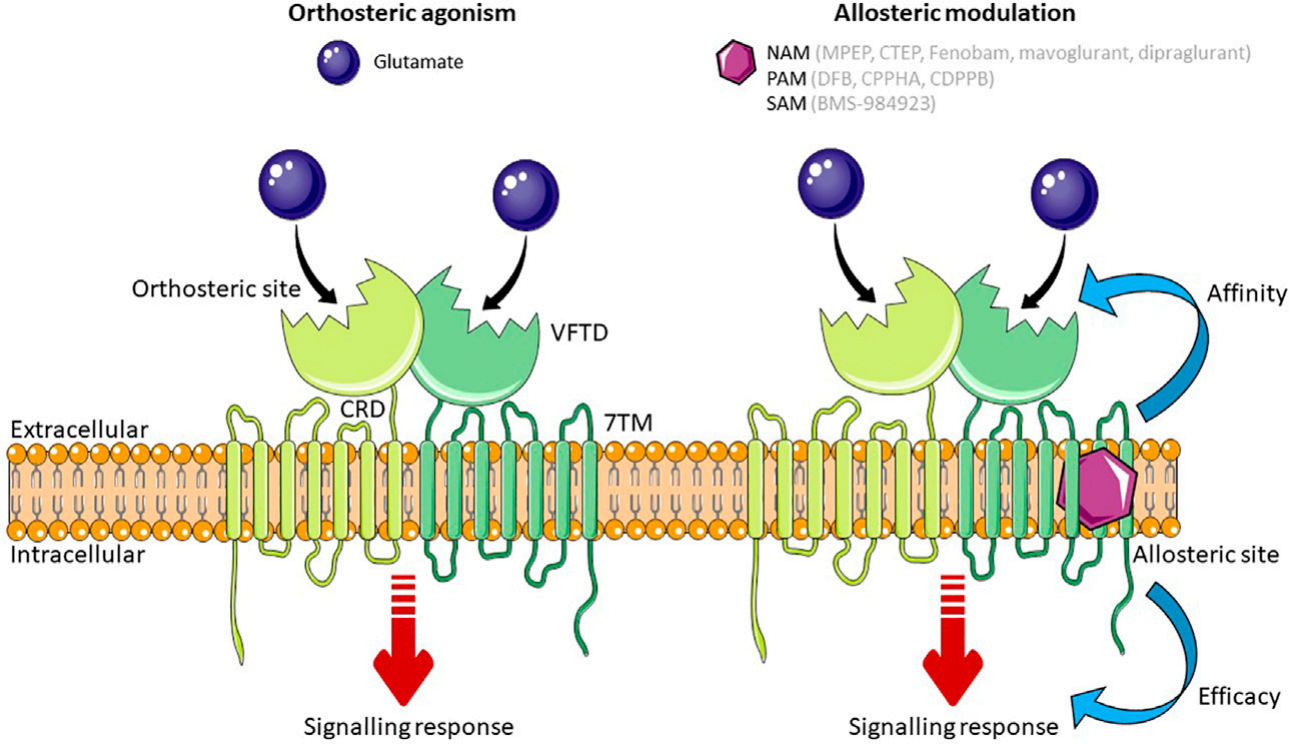
Schematic of mGlu_5_ activation. Glutamate, the endogenous ligand for mGlu_5_, binds at the orthosteric site in the VFTD. Allosteric modulators (NAMs, PAMs and SAMs) bind to a topographically distinct site in the 7TM domain. Simultaneous binding of an orthosteric agonist and an allosteric modulator effects the affinity or efficacy of the orthosteric agonist. Some allosteric modulators are able to exert their effect independent to the orthosteric agonist. Abbreviations: NAM, Negative Allosteric Modulator; PAM, Positive Allosteric Modulator; SAM, Silent Allosteric Modulator; VFTD, Venus Flytrap Domain; 7TM, 7 transmembrane domain; CRD, Cysteine Rich Domain. Figure created using Reactome Icon Library, licensed under CC BY 4.0 (Sidiropoulos et al., 2017).

All GPCRs have a similar core structure composed of an extracellular N-terminal tail, seven transmembrane α-helices connected by alternating intracellular and extracellular loops (7 transmembrane domain; 7TM), and an intracellular C-terminal tail (Rosenbaum, Rasmussen and Kobilka, 2009). Like all mGlu receptors, mGlu_5_ is organized as a dimer and has a large extracellular N-terminus (Mølck et al., 2014). This N-terminal tail is arranged as a Venus Flytrap Domain (VFTD) which forms the orthosteric binding site, where the endogenous ligand, glutamate, binds. The VFTD is connected to the 7TM by a cysteine-rich domain (Nasrallah et al., 2021) (Figure 1). In addition to binding to the orthosteric site, ligands can bind to sites that are topographically distinct from the orthosteric site, known as the allosteric site. Both group 1 mGlu receptors differ from other mGlu receptors in that they have three hydrophilic residues at the top of their allosteric binding site, which could form a bond with ligands that are group 1 specific. In mGlu_5_ specifically, there is a deep and narrow sub-pocket that is thought to be a key binding site for achieving mGlu_5_ selectivity (Harpsøe et al., 2015). Computational modelling has shown that 6 non-conserved and 4 conserved residues in the binding pocket of group 1 mGlu receptors are essential for subtype selectivity, with the non-conserved residues playing a role in ligand binding through spatial conformation (Fu et al., 2020). Using computational techniques to provide three-dimensional protein structures has paved the way for structure-based drug discovery (SBDD), a powerful method for identifying drug candidates. A highly selective ligand for mGlu_5_, HTL14242, was discovered using SBDD techniques (Christopher et al., 2015). For a detailed review on mGlu_5_ structure and its importance for the development of allosteric modulators, see Bennett et al. (2020).

Following binding of an agonist, mGlu_5_ receptors preferentially couple to the heterometric G protein Gα_q/11_ resulting in the release of Ca^2+^ from intracellular stores via downstream second messengers. Gα_q/11_ stimulates phospholipase C which cleaves phosphatidylinositol 4,5, biophosphate into diacylglycerol (DAG) and inositol 1,4,5 triphosphate (IP3). IP3 triggers the release of Ca^2+^ from intracellular stores by opening ligand-gated inositol phosphate receptors on the endoplasmic reticulum. Together with DAG, Ca^2+^ contributes to the activation of protein kinase C (PKC) (Dhami and Ferguson, 2006). As well as coupling to G proteins, GPCRs can signal *via* β-arrestins such as β-arrestin2 (Stoppel et al., 2017). Although there is no evidence for direct coupling of mGlu_5_ receptors to β-arrestins, it has been demonstrated that mGlu_5_ and β-arrestin co-immunoprecipitate (Eng et al., 2016) and that β-arrestin is necessary for some of the downstream signalling from mGlu_5_ (Stoppel et al., 2017). Stimulation of group 1 mGlu receptors can result in the activation of other downstream effector enzymes, such as extracellular regulated kinase (ERK), protein kinase B (Akt), and mammalian target of rapamycin (mTOR) (Ribeiro FM. et al., 2014).

The activity of mGlu_5_ can be pharmacologically manipulated by ligands that bind to either the orthosteric site or allosteric binding sites. The former either mimic the actions of glutamate by activating (agonists) or blocking receptor activity (antagonists). However, the orthosteric binding site is highly conserved between mGlu receptor subtypes (Wellendorph and Bräuner-Osborne, 2009), and so targeting an allosteric site within the 7TM domain may be more therapeutically beneficial in disease (Harpsøe et al., 2015) in part due to increased subtype-specific selectivity (May and Christopoulos, 2003). Moreover, allosteric modulators are able to induce diverse changes in GPCR signaling and can be designed to produce a biased signaling response, effecting therapeutically beneficial signaling pathways and not pathways that may cause adverse effects (Trinh et al., 2018). There are three categories of allosteric modulator: those that affect the binding affinity of the orthosteric ligand; those that affect the efficacy of the orthosteric ligand; and those that exert their effect independent of the orthosteric ligand (Langmead and Christopoulos, 2006). The affinity or efficacy of the orthosteric ligand can be altered in positive, negative, or neutral direction. Positive allosteric modulators (PAMs) increase agonist affinity and/or efficacy, negative allosteric modulators (NAMs) decrease agonist affinity and/or efficacy, and silent allosteric modulators (SAMs) have no effect on the affinity and/or efficacy of the orthosteric ligand, but act as a competitive agonist at allosteric sites, thus blocking PAM or NAM activity. Modulating the effect of the endogenous ligand at the orthosteric site is an advantage of allosteric ligands as exerting an effect only when and where glutamate is present means that signaling is altered in proportion to the physiological response (Trinh et al., 2018). In addition, glutamate shows neutral affinity cooperativity with ligands that bind to the allosteric site of mGlu_5_, meaning that high concentrations of glutamate do not affect the action of mGlu_5_ allosteric modulators, providing them with an advantage over orthosteric ligands which compete with glutamate (Bennett et al., 2020).

### Allosteric Modulator Drug Discovery

Several ligands which modulate the activity of mGlu_5_ receptors have been developed and progressed into clinical trials for neurodegenerative disorders and in other disorders characterised by loss of brain immune function, such as mood disorders, or disorders where addiction has formed aberrant and strong cue-based associations linked to increased craving and drug use (Table 1). These disorders are not the focus of this review, however these data are relevant to mGlu_5_ as a target to treat neurodegenerative diseases as these neuropsychiatric symptoms can overlap with neurodegenerative disorders (Husain, 2017).

**TABLE 1:** Allosteric modulators of mGlu_5_ that have entered clinical trials for neurodegenerative and neurological disorders. All clinical trials found at www.clinicaltrials.gov. For detailed *in vitro* pharmacological characterization of clinically tested mGlu_5_ NAMs see Arsova et al. (2020).

Mode of Action	Ligand	Potential therapeutic indications	Clinical trials
NAM	Mavoglurant (AFQ056)	FXS (Jacquemont et al., 2011; Levenga et al., 2011)	Completed Phase I: NCT01482143 (FXS)
		L-dopa-induced dyskinesia in PD (Berg et al., 2011; Kumar et al., 2015)	Active Phase II: NCT02920892 (FXS)
		OCD (Rutrick et al., 2017)	Terminated Phase II:
			NCT01813019 (OCD)
			NCT01433354 (FXS)
		HD (Reilmann et al., 2015)	NCT01348087 (FXS)
			NCT01019473 (HD)
			Completed Phase II:
			NCT01491932, NCT01491529, NCT01385592, NCT00888004, NCT00582673, NCT01173731, NCT01092065, NCT00986414 (PD, dyskinesias and movement disorders)
			NCT01357239, NCT01253629, NCT00718341 (FXS)
			NCT03242928 (CUD)
	Dipraglurant	L-dopa-induced dyskinesia in PD (Bezard et al., 2014)	Recruiting Phase II and III:
			NCT05116813, NCT04857359 (PD, dyskinesias and movement disorders)
			Completed Phase II: NCT01336088 (PD)
	Basimglurant (RG7090, RO4917523)	Mood disorders Lindemann et al. (2015), Fuxe and Borroto-Escuela (2015)	Completed Phase I:
			NCT02433093 (MDD)
			NCT01873508, NCT01368926, NCT01483469 (Healthy volunteers)
		FXS Youssef et al. (2018)	Completed Phase II:
			NCT01517698, NCT01750957, NCT01015430 (FXS)
			NCT01437657 (MDD)
			NCT00809562 (Depression)
	STX107	FXS	Completed Phase I:
			NCT00965432 (FXS)
			Suspended Phase II:
			NCT01325740 (FXS)
	Fenobam	FXS (Berry-Kravis et al., 2009)	Completed Phase I: NCT01806415 (Healthy volunteers)
		Anxiety disorders (Pecknold et al., 1982; Palucha and Pilc, 2007)	
		L-dopa-induced dyskinesia in PD (Rylander et al., 2010; Ko et al., 2014)	
	HTL14242	Neurological indications (Christopher et al., 2015)	Completed Phase I: NCT04462263, NCT03785054 (Healthy volunteers)
		RGH-618	Anxiety disorders	In clinical development (Sartori and Singewald, 2019)
			VU0424238	Neurological indications (Felts et al., 2017)	Selected for clinical evaluation (Felts et al., 2017)
			Raseglurant (ADX-10059)	Migraine Marin and Goadsby et al. (2010)	Terminated Phase II: NCT00820105 (Migraine)
			AZD2066	Mood disorders (Jaso et al., 2017)	Completed Phase I: NCT00686504, NCT00766012 (Healthy volunteers)
	Terminated Phase II: NCT01145755 (MDD)			
			AZD2516	Neuropathic pain	Completed Phase I: NCT00892944 (Healthy volunteers)
SAM	BMS-984923	AD (Haas et al., 2017)	Recruiting Phase I: NCT04805983 (AD)

AD, Alzheimer’s disease; CUD, Cocaine Use Disorder; FXS, Fragile X Syndrome; HD, Huntington’s disease; MDD, Major Depressive Disorder; OCD, Obsessive Compulsive Disorder; PD, Parkinson’s disease.

The first NAMs reported to be selective for mGlu_5_ were SIB-1757 and SIB-1893 (Varney et al., 1998). Structural rearrangement of these compounds led to the discovery of 2-methyl-6-(phenylethynyl) pyridine (MPEP) (Gasparini et al., 1999). Although MPEP is highly selective for mGlu_5_ as compared to other mGlu receptor subtypes, it also acts as a weak NMDA receptor antagonist (O’leary et al., 2000) and mGlu_4_ PAM (Mathiesen et al., 2003). NAMs for mGlu_5_ have been developed to show both increased selectivity and potency, including 3-[(2-methyl-1,3-thiazol-4-yl)ethynyl] pyridine (MTEP) (Cosford et al., 2003), and 2-chloro-4-((dimethyl-1-(4-(trifluoromethoxy)phenyl)-1H-imidazol-4yl)ethynyl)pyridine (CTEP), the latter of which has the highest potency and selectivity (Lindemann et al., 2011). Another potent, selective NAM for mGlu_5_ is fenobam, which was developed in the 1970’s but only found to target mGlu_5_ in 2005 (Porter et al., 2005). As well as acting as an antagonist, fenobam has inverse agonist activity, meaning it not only antagonises agonist activity, but also exerts the opposite effect by inhibiting constitutive receptor activity. Fenobam acts via the same allosteric binding site as MPEP and MTEP within in the 7TM between TM 3, 6 and 7 (Gregory et al., 2011).

NAMs of mGlu_5_ have shown promising results in preclinical models but many have progressed to phase II clinical trials for neurodegenerative diseases where they have failed to show efficacy in, including mavoglurant, basimglurant, STX107, fenobam, raseglurant, and AZD 2066 (Table 1). Mavoglurant met its primary endpoint in a phase II trial for cocaine use disorder and is moving into phase III (clinicaltrials.gov: NCT03242928). It was shown to reduce neuropsychiatric symptoms of addiction, with a significant reduction in the cocaine use in the group that received mavoglurant compared to patients receiving the placebo. One mGlu_5_ NAM, dipraglurant, has shown efficacy in phase II trials in neurodegenerative disease, specifically for levodopa-induce dyskinesia in patients with Parkinson’s disease (clinicaltrials.gov: NCT01336088). It has recently progressed to phase III (clinicaltrials.gov: NCT04857359).

The first mGlu_5_ PAM to be identified was 3,3′-difluorobenzaldazine (DFB) (O’Brien et al., 2003). DFB had low potency, and a year later N-[5-chloro-2-[(-1,3-dioxoisoindolin-2-yl)methyl]phenyl]-2-hydroxybenzamide (CPPHA), which exhibited greater potency, was identified (O’Brien et al., 2004). The first PAM developed with enough solubility to allow for *in vivo* studies was 3-cyano-N-(1,3-diphenyl-1H-pyrazol-5-yl)benzamide (CDPPB) (O’Brien et al., 2003, 2004; Lindsley et al., 2004). DFB and CDPPB both bind to the MPEP binding site in the seven transmembrane domain, whereas CPPHA binds to a distinct allosteric site.

The mGlu_5_ SAM BMS-984923 was identified in an attempt to identify mGlu_5_ PAMs for schizophrenia (Huang et al., 2016). BMS-984923 showed a therapeutic benefit in a preclinical mouse model of Alzheimer’s disease and is currently in a phase I clinical trial to determine its safety and tolerability in human Alzheimer’s disease patients (clinicaltrials.gov: NCT04805983).

## Therapeutic Potential in Neurodegenerative Diseases

This review will discuss current evidence that highlights the potential of mGlu_5_ allosteric modulators to treat neurodegenerative diseases, including Alzheimer’s disease, Huntington’s disease, amyotrophic lateral sclerosis, and Parkinson’s disease. Additionally, this review will explore the role of mGlu_5_ receptors in neuroinflammatory responses, and the potential for this GPCR to modulate neuroinflammation. Neurodegenerative diseases share a number of common pathologies, including neuroinflammation, and so a ligand which targets mGlu_5_ on inflammatory cells, such as astrocytes and microglia, could have a therapeutic benefit across neurodegenerative diseases. However, as discussed below in this review, this is complicated by uncertainty surrounding the relationship between inflammatory cells types, their relative contribution to neurodegeneration, and whether they should be up or downregulated in order to be therapeutically beneficial.

## Alzheimer’s Disease

Alzheimer’s disease (AD) is a progressive neurodegenerative disease, characterised by decline in cognitive function with attention, episodic memory, and executive function domains most affected (Giorgio et al., 2020). The disease threatens society with a substantial health and economic burden due to its increasing prevalence in line with an ageing population, and the lack of preventative treatments (Qiu et al., 2009); most current treatments for AD are symptomatic rather than disease-modifying in nature (Yiannopoulou & Papageorgiou, 2013).

The neuropathology of AD is characterized by the presence of extracellular amyloid plaques and intracellular neurofibrillary tangles (Deture and Dickson, 2019). Early AD research focused on plaques, composed primarily of β-amyloid (Aβ), as the neurotoxic species. Aβ also exists in the brain in the form of soluble Aβ oligomers, and it is thought that these oligomers are the predominant source of neurotoxicity in the Alzheimer’s brain, rather than the plaques themselves (Kayed and Lasagna-Reeves, 2013). Aβ oligomers have been shown to bind with high affinity to cellular prion protein (Laurén et al., 2009), a complex requiring mGlu_5_ as a co-receptor and resulting in the potentially neurotoxic release of intracellular Ca^2+^ (Um et al., 2013). This implicates mGlu_5_ as playing a role in Aβ oligomer-related pathology. For a detailed review on aberrant mGlu_5_ signaling following binding of Aβ oligomers see (Abd-Elrahman et al., 2022).

The genetic deletion or pharmacological blockade of mGlu_5_ have been shown to be neuroprotective in rodent models of AD. Genetic deletion of mGlu_5_ in the APPswe/PS1∆9 (APPswe) mouse model of AD was shown to reverse memory deficits in mice at both 9 and 12 months of age using a Morris Water Maze paradigm (Hamilton et al., 2014). Similarly, blockade of mGlu_5_ with either MTEP or CTEP improved learning and memory deficits in both APPswe and 3xTg mice mouse models (Um et al., 2013; Hamilton et al., 2016). In addition to improvements in spatial memory deficits, the chronic administration of CTEP was observed to correct deficits in episodic and recognition memory, both deficits that appear early in the disease progression of APPswe mice, similar to the early clinical presentation of AD (Hamilton et al., 2016). Strikingly, these studies found that both genetic deletion and pharmacological blockade of mGlu_5_ reduced the presence of AD pathology, including Aβ oligomers and plaques, (Hamilton et al., 2014; Hamilton et al., 2016), and decreased synaptic loss and increased synaptic density (Um et al., 2013). These findings are of particular interest as they indicate that mGlu_5_ signalling contributes directly to the establishment of AD-like pathology in APPswe and 3xTg AD mice.

Aberrant mGlu_5_ signaling in AD mice has been observed to inhibit autophagy, the process by which cellular organelles and protein aggregates are cleared (Nixon, 2013), *via* ubiquitination and proteasomal degradation of the autophagy related 14 (ATG14) protein (Zhang et al., 2015; Abd-Elrahman et al., 2017). The reduction in AD pathology and reversal of memory deficits observed in AD mice following genetic deletion or pharmacological blockade of mGlu_5_ is paralleled by an increase in autophagy via alterations in Zinc finger and BTB domain-containing protein 16 (ZBTB16)- and Unc-51-like kinase 1 (ULK1)-dependent pathways (Abd-Elrahman et al., 2018). Optineurin, a cytosolic protein, is essential for this regulation of mGlu_5_ -dependent autophagic signalling (Ibrahim et al., 2021). This has also been observed in preclinical Huntington’s Disease mouse models, indicating that mGlu_5_ contributes to neurodegeneration via conserved mechanisms (Abd-Elrahman et al., 2017), and suggests that antagonism of mGlu_5_ represents an effective approach to reverse progression of neurodegeneration by activating autophagy.

Another therapeutic target for the treatment of AD is the M_1_ muscarinic acetylcholine receptor (mAChR) and M_1_ PAMs and a partial agonist have emerged as promising strategy for improving cognitive function in AD (Scarpa, Hesse and Bradley, 2020; Abd-Elrahman and Ferguson, 2022; Nathan et al., 2022). The addition of Aβ to rodent brain slices impairs the function of M_1_, which is reversed by the addition of MPEP or another mGlu_5_ NAM, LSN (Yi et al., 2020). The authors suggest that Aβ drives M1 dysfunction via aberrantly activating mGlu_5_. This provides another mechanism by which mGlu_5_ antagonism may be beneficial in AD: the restoration of M_1_ mAChR function.

Whilst the use of mGlu_5_ NAMs to modify disease progression in AD has been promising, disrupting glutamate signaling *via* the antagonism or genetic deletion of mGlu_5_ has deleterious effects on learning and memory independent of AD (Simonyi, Schachtman and Christoffersen, 2005). A recent study suggested that the genetic deletion of mGlu_5_ accelerated neurodegeneration, with mGlu_5_ knockout mice having increased neuronal loss, astrogliosis, and microglial activation as compared to wild-type mice (Carvalho et al., 2019). Therefore, the use of mGlu_5_ NAMs to treat neurodegeneration could have a negative impact on brain regions that do not yet show AD pathology. However, it may also be that the use of mGlu_5_ NAMs to treat neurodegeneration is dependent on the presence of Aβ and tau.

Moreover, mGlu_5_ NAMs have been found to have psychomimetic effects in humans. For example, although fenobam has shown anxiolytic-like effects in human patients, it also induced psychomimetic effects including hallucinations and insomnia (Friedmann et al., 1980; Porter et al., 2005; Abou Farha et al., 2014). This may be due to modulation of the *N*-methyl-D-aspartate receptors (NMDARs) which are coupled to mGlu_5_ both structurally and functionally. Inhibition of NMDARs induces psychomimetic effects in both rodents and humans, which may be enhanced by inhibition of mGlu_5_ (Marino and Conn, 2002). Additionally, some mGlu_5_ NAMs have inverse agonist activity, which may also contribute to psychotic side effects in human patients (Porter et al., 2005; Keck et al., 2012). These side effects may limit the therapeutic window of mGlu_5_ NAMs, although no psychotomimetic effects were seen at the efficacious dose of mavoglurant in patients with cocaine use disorders in safety and tolerability studies (Ufer et al., 2016).

It may be important to elucidate mechanisms of blocking Aβ-mediated mGlu_5_ signaling, without blocking glutamate signalling. Unlike NAMs, mGlu_5_ SAMs do not alter levels of glutamate-mediated Ca^2+^ signaling (Haas et al., 2017). Silent allosteric modulation of mGlu_5_ using the mGlu_5_ SAM BMS-984923 has been shown to reduce the interaction between Aβ oligomers and cellular prion protein, resulting in an improvement in cognitive deficits and synaptic loss in APPswe mice without an alteration in physiological glutamate signaling (Haas et al., 2017). No improvements in Aβ plaque load were observed, suggesting that BMS-984923 acted to block processes downstream of mGlu_5_, rather than altering Aβ accumulation. The treatment of these mice began after Aβ plaques, synapse loss, and memory impairment has developed, suggesting that the use of SAMs to treat AD could slow or stop disease progression in advanced stages.

Perhaps surprisingly, considering the potential for mGlu_5_ NAMs in the treatment of AD, there is evidence to suggest that the agonism of mGlu_5_ may also be neuroprotective in AD. In both sexes of T41 mice, which overexpress mutant human amyloid precursor protein, and in wild-type mice injected with Aβ aggregates, neuronal loss was prevented by treatment with the mGlu_5_ PAM CDPBB. Moreover, elevated levels of microglial and astrocytic markers observed in the T41 mice were partially reversed in the CA1 hippocampal region. However, despite the reversal in Aβ-mediated neurotoxicity, CDPBB was not able to promote cognitive improvement in the T41 mice. The authors suggest that this could be due to the age at which the mice were treated; at 14 months the animals already show severe AD pathology and cognitive decline (Bellozi et al., 2019). Interestingly, the APPswe and 3xTg mice used in the previously discussed mGlu_5_ NAM experiments were treated with MTEP or CTEP months after the development of Aβ pathology and memory deficits, yet an improvement in cognition was still observed (Um et al., 2013; Hamilton et al., 2014; Hamilton et al., 2016; Abd-Elrahman et al., 2018).

The binding of Aβ oligomers to mGlu_5_ in AD not only activates mGlu_5_, but it has also been shown to induce the clustering of mGlu_5_ at the surface of synapses (Renner et al., 2010). Increased cell surface expression of mGlu_5_ has been observed in a number of mouse AD models (Um et al., 2013; Hamilton et al., 2014; Abd-Elrahman et al., 2018). Hamilton et al. (2014), for example, observed a 4.4 fold increase in mGlu_5_ cell surface expression in APPswe mice as compared to control mice, without a change in total cellular mGlu_5_ level. However, it should be noted that a recent study using highly sensitive, ultrastructural techniques such as SDS-FRL in the same mouse model at the same time point as Hamilton and colleagues demonstrated that, although the total level of mGlu_5_ was unchanged in APPswe mice as compared to control mice, the localisation of mGlu_5_ was significantly reduced along the cell surface of certain hippocampal cells, including pyramidal cells in the CA1 region and granule cells in the dentate gyrus (Martín-Belmonte et al., 2021). In addition, PET imaging in 5xFAD mice found that mGlu_5_ was reduced in the hippocampus and striatum of diseased animals as compared to controls (Lee et al., 2018). These findings were corroborated by a human PET study showing a 43% reduction of mGlu_5_ expression in the hippocampus of patients with mild AD (Mecca et al., 2020).

In models where mGlu_5_ is recruited to the cell surface, it is thought that Aβ oligomers act as an extracellular scaffold thus reducing the lateral diffusion of mGlu_5_ receptors (Renner et al., 2010). This means that the receptor is more readily accessible for activation by both Aβ oligomers and glutamate, thereby increasing Ca^2+^ levels (Renner et al., 2010) and potentially excitotoxicity. Therefore, pharmacologically activating mGlu_5_ to treat AD could be neurotoxic in the long term. A mGlu_5_ PAM 5PAM523 given orally to wild-type rats resulted in indications of neurotoxicity, including convulsions and excitability, which occurred in an mGlu_5_-dependent manner (Parmentier-Batteur et al., 2014). Similar to mGlu_5_ NAMs, it may be that this mGlu_5_ PAM-induced toxicity is via modulation of NMDAR currents (Conde-Ceide et al., 2015). Of note, Abd-Elrahman et al. (2018) found that the elevation in cell surface expression observed in both APPswe and 3xTg mice as compared to wild-type mice was reversed in animals treated with CTEP. Chronic administration of an mGlu_5_ NAM may slow the progression of AD pathology by disrupting the Aβ oligomer-mediated increase in mGlu_5_ cell surface expression.

In humans, PET imaging has been used to investigate changes in mGlu_5_ expression in healthy patients as they age. A recent study found no significant change in mGlu_5_ expression across brain regions when their analysis was corrected to include brain atrophy with age (Mecca et al., 2021). In AD patients, PET imaging studies have shown a significant decrease in mGlu_5_ expression in the hippocampus and amygdala, which remained after correcting to brain atrophy (Mecca et al., 2020; Treyer et al., 2020). Conversely, a post-mortem study of mGlu_5_ binding saw increases in mGlu_5_ expression in the frontal cortex and hippocampus of severe AD patients as compared to controls (5.2-fold and 2.5-fold respectively) (Müller Herde et al., 2019). This study, however, was underpowered with only 2 AD patients and 4 controls assessed and no supporting demographic data to assess the degree to which patient and control samples were matched giving a high probability of a type 1 error. Notwithstanding, it cannot be ruled out that there may be variation in mGlu_5_ up-regulation at different disease-stages, with mGlu_5_ up-regulation potentially only occurring in later-stage disease. Further longitudinal research is required to fully understand changes in mGlu_5_ expression in human AD.

Further thought and investigation are needed to reconcile the findings that both mGlu_5_ NAMs and PAMs can be neuroprotective in rodent models of AD, but it may be that antagonism and agonism of mGlu_5_ are neuroprotective at different stages of disease progression. Recently, it has been suggested that the contribution of mGlu_5_ to AD neuropathology is disease-stage dependent (Abd-Elrahman KS. et al., 2020). APPswe mice were given CTEP from the age of 6 months for either 24 or 36 weeks. When administered for 24 weeks, CTEP reversed memory deficits, increased autophagy and reduced AD pathology, including Aβ plagues and neuroinflammation. However, when treatment was extended to 36 weeks, CTEP was found to be ineffective at reversing memory deficits or AD pathology. The authors suggest that this may be due to reduced contribution of mGlu_5_ to AD-related pathology at advanced disease-stages. Going forward, it will be important to study the long-term efficacy of potential therapeutic targets related to disease trajectory and known risk factors, such as APOE status (Evans et al., 2019). Taken together, these data support a role for targeting mGlu_5_ in early stages of AD, perhaps in combination with the current standard of care (i.e., acetylcholinesterase inhibitors) to slow disease progression and/or improve the treatment response to the standard of care.

Most research into the effect of mGlu_5_ antagonism on AD mouse models has used male mice. However, recent investigations into whether the observed changes are conserved in female AD mice have suggested that mGlu_5_ may not make a significant contribution to AD pathology in female AD mice (Abd-Elrahman KS. et al., 2020). As discussed, Aβ oligomers bind to mGlu_5_ in a complex with cellular prion protein. Using radioligand binding assays, it was demonstrated that this complex formed only in the brains of male, but not female, mice. The same finding was observed in human brain tissue. Moreover, treating primary cortical neurons with an mGlu_5_ agonist or with Aβ oligomers only activated neuroprotective autophagy pathways in neurons derived from male, but not female, mouse embryos. Taken together, these data indicate that there are sex-specific differences in mGlu_5_ receptor signaling. In addition, it was found that the cell surface expression of mGlu_5_ differed between male and female mice. As previously mentioned, Aβ oligomers promote the clustering of mGlu_5_ receptors at the cell surface, however Abd-Elrahman and colleagues found that this increase in cell surface expression occurred only in male APPswe mice. Conversely, there was little mGlu_5_ cell surface expression in the cortex and hippocampus of female APPswe mice. Both sexes of APPswe mice have comparable levels of Aβ oligomers, neuroinflammatory markers, and cognitive decline, however chronic treatment of these mice with CTEP was found to improve cognition and reduce AD pathology in male mice only. These findings provide evidence that the contribution of mGlu_5_ receptors to AD-related neuropathology in APPswe mice is sex-dependent. This must be taken into consideration when investigating the potential of mGlu_5_ as a drug target for AD, and perhaps other diseases, too.

## Huntington’s Disease

Huntington’s disease (HD) is an autosomal dominant neurodegenerative disease characterized by motor deficits including chorea and loss of coordination, cognitive decline, and psychiatric changes that ultimately results in death (McColgan and Tabrizi, 2018). Like Alzheimer’s disease, there are currently no disease-modifying treatments (McColgan and Tabrizi, 2018).

HD is caused by an inherited CAG trinucleotide repeat expansion in the gene that codes for the huntingtin protein (Htt), resulting in a mutant version of the protein that has an abnormally long polyglutamine repeat (mHtt) (MacDonald et al., 1993). This elongated protein can be cleaved into fragments which then aggregate to form neuronal intranuclear inclusions (NII), the presence of which correlates with disease progression (Miller et al., 2010).

The most striking pathological feature of Huntington’s disease is the loss of striatal neurons, although degeneration is not confined to these cells (Masnata & Cicchetti, 2017). There is high expression of mGlu_5_ in the striatum (Shigemoto et al., 1993) where it is enriched in medium-sized striatal cells (Testa et al., 1995). It has been shown that mGlu_5_ interacts directly with both Htt and mHtt, and that this interaction uncouples mGlu_5_ receptor signaling (Anborgh et al., 2005) and leads to increased intracellular Ca^2+^ levels, which is associated with excitotoxic cell death (Tang et al., 2003). This dysregulation of Ca^2+^ signalling is a feature of HD mouse models (Ribeiro et al., 2010). In addition, mGlu_5_ is thought to play a role in motor control as mGlu_5_ knockout mice have increased locomotor activity (Ribeiro F. M. et al., 2014). Taken together, these observations suggest that mGlu_5_ is a potential target in the treatment of Huntington’s disease.

Longitudinal PET imaging studies carried out in the *zQ*175 HD model showed a significant reduction in mGlu_5_ binding in the striatum and cortex as compared to wild-type mice over time (Bertoglio et al., 2018). There was no significant decrease in neuronal density in these mice, suggesting that the reduction in mGlu_5_ was not due to neuronal loss. Examination of post-mortem human HD tissue showed a decrease in mGlu_5_ expression in the caudate and putamen. However, as neuronal cell density was also significantly reduced, it may be that the reductions observed were due to neuronal loss (Gulyás et al., 2015). To date, there is no *in vivo* mGlu_5_ PET imaging data available in human HD patients to assess these changes. Notwithstanding, there are *in vivo* indications of altered cholinergic function in HD which may be related to altered mGlu_5_ function as previously discussed (D’Souza and Waldvogel et al., 2016). This is an important area of research to understand novel treatment strategies for this disease.

It has been shown that the blockade of mGlu_5_ is neuroprotective in mouse models of HD. Treatment with the mGlu_5_ NAM MPEP was shown to slightly increase survival and reverse the loss of motor coordination in R6/2 HD mice. However, this was not paralleled by a reduction in NII formation. Interestingly, mice treated with MPEP were observed to have larger NIIs in their cortical neurons (Schiefer et al., 2004). Other studies have found the neuroprotective effects of mGlu_5_ blockade to be paralleled by a reduction in NII formation. The genetic deletion of mGlu_5_ in Hdh^Q111/Q111^ HD mice improved motor coordination and reduced the formation of NIIs (Ribeiro FM. et al., 2014). Likewise, the chronic administration of CTEP to 12-month-old *zQ175* huntingtin knockin (*zQ175*) mice improved both motor and cognitive deficits, and significantly reduced mHtt aggregates and neuronal cell death (Abd-Elrahman et al., 2017). Interestingly, this study found that acute blockade of mGlu_5_ (1 week) was effective at ameliorating motor and cognitive deficits in heterozygous, but not homozygous, *zQ175* mice.

A key regulator of HTT-mediated gene expression is the repressor element 1-silencing transcription factor/neuron restrictive silencer factor (REST/NRSF) (Rigamonti et al., 2009). In HD, REST/NRSF leads to the decreased transcription of several neuronal genes (such as brain-derived neurotrophic factor, BDNF) in the nucleus of diseased cells. Using primary neuronal cultures, mGlu_5_ was shown to regulate REST/NRSF expression via modulating N-cadherin/β-catenin interactions (De Souza et al., 2020); the activation of mGlu_5_ with 3,5-dihydroxyphenylglycine (DHPG) resulted in increased REST/NRSF expression, whilst the inhibition of mGlu_5_ with CTEP resulted in decreased REST/NRSF expression. This was paralleled by changes in SNAP-25 expression. These findings were also observed *in vitro* using *zQ175* mice chronically treated with CTEP, and in BACHD mGlu_5_ KO mice, confirming that mGlu_5_ modulates REST/NSRF signaling in HD. The authors suggest that the improvements in motor function and reduction in disease pathology after the pharmacological and genetic blockade of mGlu_5_ in HD mouse models may be due to a reduction in aberrant mGlu_5_ -regulated REST/NRSF signaling.

Similar to AD, it has been suggested that mGlu_5_ antagonism may improve cognitive function in HD mice by promoting the increased removal of aggregated mHtt *via* autophagy. The presence of mHtt aggregates correlates highly with HD progression, and the number and size of these aggregates is reduced *via* mGlu_5_ deletion or antagonism in HD mice models (Ribeiro F. M. et al., 2014; Abd-Elrahman et al., 2017). This observation is linked to increased autophagy *via* the same ZBTB16- and ULK-1-dependent mechanisms observed to be altered in AD (Abd-Elrahman et al., 2017, 2018). The chronic antagonism of mGlu_5_ promotes a reduction in ZBTB16 expression, which, in turn, leads to the rescue of ATG14, a key autophagy adapter. In addition, mGlu_5_ inhibition reduces the inhibitory phosphorylation of ULK1, resulting in its activation which is essential for the phosphorylation of ATG13 (Abd-Elrahman et al., 2017). This ULK-1 activation is triggered by the normalization of aberrant mammalian target of rapamycin (mTOR) activation (Abd-Elrahman and Ferguson, 2019). ATG13 is required for the formation of the autophagosome, which is a critical autophagy process (Abd-Elrahman and Ferguson, 2019). Therefore, targeting mGlu_5_ with NAMs represents an effective approach to slow the progression of HD by promoting autophagy to reduce the aggregation of mHtt aggregates.

Apoptosis is another process that is altered by mHtt (Steffan et al., 2000). Chronic mGlu_5_ antagonism in partially reduced neuronal apoptosis, and increased cell survival in *zQ*175 mice (Abd-Elrahman et al., 2017). Further examination of these mice revealed that chronic CTEP administration enhanced CREB-dependent BDNF transcription, which reduces apoptosis and promotes neuronal survival (Abd-Elrahman et al., 2019). It is likely that the promotion of autophagy to clear mHtt facilitates this increase in CREB expression and BNDF production; this can be confirmed by blocking autophagy in measuring BNDF synthesis in a HD mouse model. Taken together, these findings suggest that targeting mGlu_5_ with a NAM may be beneficial in treating neurodegeneration by promoting autophagy and reducing apoptotic neuronal cell loss.

As in AD, the effects of mGlu_5_ antagonism on HD pathology have been studied predominantly in male HD mice. Recent work compared the response of male and female *zQ*175 mice to chronic treatment with CTEP (Li et al., 2022). This study showed that improvements in motor and cognitive skills differed between the sexes. Chronic mGlu_5_ antagonism improved the motor performance of male HD mice in grip strength and rotarod tests after both 4 and 12 weeks of CTEP administration. In comparison, female mice only saw an improvement in rotarod performance after 12 weeks of treatment. In regards to cognition, only male HD mice regained their cognitive ability in a novel object recognition test. Despite differences in motor and cognitive improvements, both sexes of HD mice had an attenuation of HD pathology, including reduced mHtt aggregates and neuronal cell death. These findings suggest that antagonism of mGlu_5_ could be neuroprotective in both male and female rodents, but further work is required to understand any differences in the efficacy of mGlu_5_ NAMs between the sexes.

The agonism of mGlu_5_ using PAMs may also be neuroprotective in the treatment of HD. DFB, CDPPB, and VU1545 were shown to be neuroprotective in primary striatal culture, all preventing excitotoxic cell death caused by elevated glutamate or NMDA concentrations (Doria et al., 2013). The chronic administration of CDPBB has been shown to reverse motor and cognitive deficits in the BACHD mouse model of HD (Doria et al., 2013, 2015). This cognitive improvement is paralleled by the increased activation of neuroprotective pathways, including Akt, ERK1/2, and BDNF expression, and a reduction in the formation of mHtt aggregates (Doria et al., 2015). Sub-chronic administration (8 days) of another mGlu_5_ PAM, VU0409551, was shown to ameliorate memory impairments in the same HD mouse model (Doria et al., 2018). These mice had a significant increase in mGlu_5_ cell surface expression, especially in the hippocampus and striatum, and showed an increase in the regulation of several genes that are known to play a role in synaptic plasticity, such as BDNF, c-Fos and PSD95. BACHD mice exhibit motor and cognitive impairments from 6 months of age, yet mHtt aggregates and neuronal cell death are only apparent at 12 months of age. In the studies mentioned here, mice were treated after the appearance of behavioral deficits, but before the appearance of HD neuropathology. Thus, the authors suggest that CDPPB and VU0409551 may enhance the memory of BACHD mice independent of a neuroprotective effect, by activating synaptic plasticity pathways (Doria et al., 2018).

The signalling of mGlu_5_ is altered in neuronal cultures and brain slices from pre-symptomatic Hdh^Q111/Q111^ HD mice, with mGlu_5_ agonism leading to a reduction in mGlu_5_ -mediated IP3 formation due to an increase in PKC-mediated mGlu_5_ receptor desensitisation (Ribeiro et al., 2010). This desensitisation may be neuroprotective, resulting in a reduction in toxic Ca^2+^ signaling. Moreover, mGlu_5_ activation in these cultures resulted in an increase in Akt and ERK activation, both of which can be neuroprotective. These observations were present only in pre-symptomatic Hdh^Q111/Q111^ HD mice, and it may be that the neuroprotection provided by mGlu_5_ agonism may be lost at later disease stages. Moreover, despite a reduction in IP3 formation after mGlu_5_ agonism in the diseased neuronal cultures, the authors saw an increase in Ca^2+^ release, which suggests that the neuroprotective benefit of mGlu_5_ agonism in these cells may not be sufficient to reduce excitotoxic levels of Ca^2+^ signaling.

## Amyotrophic Lateral Sclerosis

Amyotrophic Lateral Sclerosis (ALS) is a fatal neurodegenerative disease characterized by the progressive loss of motor neurons in the motor cortex, brainstem, and spinal cord which results in progressive weakness and muscle atrophy (Mejzini et al., 2019). Most cases of ALS are sporadic, but around 10% of affected individuals have familial ALS which is inherited in an autosomal dominant manner (Kirby et al., 2016). The most widely used treatment for ALS, Riluzole, is symptomatic in nature, rather than working to halt disease progression (Mejzini et al., 2019). Riluzole modulates the glutamatergic system, but its effects are modest and wear off over time (Cetin et al., 2015), and thus targeting mGlu_5_ may be a more effective strategy.

Altered excitatory neurotransmission plays a key role in the progression of ALS. *SOD1* ^*G93A*^ mice and human ALS patients have increased concentrations of extracellular glutamate in their spinal cord plasms and cerebrospinal fluid (Shaw et al., 1995; Alexander et al., 2000; Wuolikainen et al., 2011). Different mechanisms by which glutamate levels are sustained have been proposed, such as an increased glutamate release into, and insufficient clearance of glutamate from, the synaptic cleft (King et al., 2016). In *SOD1* ^*G93A*^ mice, mGlu_5_ activation results in the abnormal release of glutamate which occurs in both pre-symptomatic and late-stage disease (Giribaldi et al., 2013; Bonifacino et al., 2019). Moreover, mGlu_5_ expression is increased in the brain of *SOD1* ^*G93A*^ mice as compared to wild-type mice. This upregulation is observed in the hippocampus, striatum, cortex, and spinal cord and is found to increase in line with disease progression in the hippocampus, spinal cord and cortex (Giribaldi et al., 2013; Brownell et al., 2015; Bonifacino et al., 2019). Autoradiography studies in human post-mortem ALS brain tissue showed that mGlu_5_ was upregulated in diseased brains as compared to controls, particularly in the motor, frontal and temporal cortices and basal ganglia (Müller Herde et al., 2019).

Reducing mGlu_5_ expression in ALS *SOD1* ^*G93A*^ mice by crossing the animals with mice heterozygous for the mGlu_5_ knockout mutation (*Grm5* ^*+/−*^) has been shown to delay disease onset and prolong survival. This was paralleled by a reduction in a number of histological characteristics; motor neurons were preserved and astrogliosis and microgliosis were reduced (Bonifacino et al., 2017). Complete knockout of mGlu_5_ in *SOD1* ^*G93A*^ mice (*SOD1* ^*G93A*^/*Grm5* ^*−/−*^) resulted in an enhanced improvement in delayed disease onset and survival (Bonifacino et al., 2019). In *SOD1* ^*G93A*^/*Grm5* ^*+/−*^ mice, with reduced mGlu_5_ expression, motor improvements were observed solely in male mice. When mGlu_5_ was completely ablated, no difference was seen between male and female mice; both sexes showed significant improvements in motor coordination and muscle strength, although the improvements seen in the male mice were slightly more pronounced. Perhaps confusingly, the same group treated *SOD1* ^*G93A*^ mice with the mGlu_5_ NAM CTEP and found that low doses improved motor skills and prolonged survival in female mice only. With an increased CTEP dose, improvements were seen in both sexes, but with improvements in the female mice being more pronounced (Milanese et al., 2021). Why genetic blockade of mGlu_5_ and blockade with a NAM showed differences in sex-specific responses requires further thought. Also of note, similar to findings in AD models (Abd-Elrahman K. S. et al., 2020), the authors found that CTEP had a disease-stage dependent effect, with improvements in specific motor skills (muscle force) being seen during early disease stages only. Despite complexity surrounding sex- and disease-stage specific effects of mGlu_5_ antagonism on ALS, mGlu_5_ NAMs represent a potentially promising avenue for ALS future research.

## Parkinson’s Disease

Parkinson’s disease (PD) is a progressive neurodegenerative disease characterized by the death of dopaminergic neurons in the substantia nigra and the abnormal accumulation of the intracellular protein α-synuclein. Clinically, patients display motor symptoms such as bradykinesia (slow movements), shaking, and rigidity, and non-motor symptoms such as difficulty sleeping, mood disorders, and dementia (Poewe et al., 2017). There is currently no cure, but the most effective drug for treating PD is dopamine-replacement therapy using L-3,4-dihydroxyphenylalanine (L-DOPA). However, the long-term use of L-DOPA is associated with L-DOPA-induced dyskinesia (LID) which involves movement disorders such as chorea and dystonia (Thanvi, Lo and Robinson, 2007).

It is well documented that increased glutamate signaling, particularly in the basal ganglia where mGlu_5_ is highly expressed, is associated with the neuropathology of PD and LID (DeLong and Wichmann, 2015). It has been suggested that the mGlu_5_ NAMs might be an effective mechanism by which excessive glutamate transmission in PD could be decreased. As previously discussed, cellular prion protein has been shown to act as a co-receptor for mGlu_5_ and Aβ oligomers (Laurén et al., 2009). Similarly, α-synuclein oligomers have been observed to interact physically with cellular prion protein via mGlu_5_. This interaction phosphorylates Fyn kinase and results in the subsequent release of intracellular calcium (Ferreira et al., 2017), thus implicating mGlu_5_ as playing a role in α-synuclein oligomer-related pathology.

The two most widely used PD animal models are the 6-hydroxydopamine (6-OHDA)-lesioned model, which involves destroying nigrostriatal dopaminergic neurons by unilaterally injecting 6-OHDA into the forebrain (Simola, Morelli and Carta, 2007), and the 1-methyl-4-phenyl-1,2,3,6-tetrahydropyridine (MPTP)-lesioned model, which involves the administration of MPTP, a neurotoxin that causes an acute loss of striatal dopamine neurons (Meredith and Rademacher, 2011; Porras et al., 2012). A large number of studies have demonstrated that mGlu_5_ antagonists are able to ameliorate motor symptoms in these animal models of PD.

Early studies showed that the antagonism of mGlu_5_ was able to reduce motor deficits in PD animal models, with the acute administration of MPEP resulting in an attenuation in unilateral rotating behavior in the rat 6-OHDA-lesioned model (Spooren et al., 2000). The effect seen was small, and the authors suggested that mGlu_5_ antagonists may not be sufficiently effective to use alone in the treatment of PD. v (Breysse et al., 2002).

The administration of MPEP, and the genetic deletion of mGlu_5_, have both been shown to reduce nigrostriatal damage and increase survival in MPTP-lesioned rats (Battaglia et al., 2004). Furthermore, the chronic administration of MPEP improves non-motor deficits, including working and recognition memory in preclinical rodent models (Hsieh et al., 2012). This improvement in cognition was paralleled by a reduction in lesion-induced dopaminergic degeneration. Similar results have been observed in MPTP-lesioned monkeys after L-DOPA and acute or chronic MPEP treatment (Morin et al., 2010; Morin et al., 2013a). Strikingly, MPTP-lesioned monkeys treated with both MPEP and L-DOPA developed 72% less dyskinesia compared to those treated with L-DOPA alone (Morin et al., 2013b).

Similarly, acute administration of MTEP has been observed to maintain anti-parkinsonian effects in L-DOPA-treated MPTP-lesioned monkeys and reduced peak dose LID by 96% (Johnston et al., 2010). Moreover, monkeys treated with both MPTP and MTEP for 18–21 weeks did not develop parkinsonian symptoms compared to monkeys treated with MPTP alone (Masilamoni et al., 2011). Furthermore, acute MTEP administration reduced catalepsy and muscle rigidity in a rat model of parkinsonism (Ossowska et al., 2005).

The 6-ODHA model of PD is thought to be more representative of the degeneration that occurs in human PD patients, as the loss of dopaminergic neurons occurs more slowly than in MPTP models (Schober, 2004). Knockout mGlu_5_ mice showed a reduction in 6-ODHA-induced neuronal loss, similar to findings in MPTP-lesioned rats (Black et al., 2010; Battaglia et al., 2004). Moreover, 6-ODHA-lesion mGlu_5_ knockout mice performed better than wild-type mice at certain motor tests, such as the pole and motor asymmetry tests. The inhibition of mGlu_5_ using CTEP in this model showed a duration-dependent improvement in motor deficits, with a partial reversal in deficits seen after 1 week of administration, and a complete attenuation of certain deficits, such as coordination, after 12 weeks of treatment (Farmer et al., 2020). These improvements were paralleled by the activation of the mTOR pathway, an increase in striatal BDNF levels, and the re-innervation of dopaminergic terminals in the striatum. The administration of other mGlu_5_ NAMs, including MPEP, fenobam, mavoglurant and dipraglurant, have been shown to reduce LID severity and enhance the anti-parkinsonian effects of L-DOPA treatment in 6-OHDA-lesioned rats (Rylander et al., 2010; Huang et al., 2018).

PET imaging in human PD patients has shown that mGlu_5_ binding is slightly increased in the brains of PD patients, specifically in the putamen, hippocampus, and amygdala (Kang et al., 2019). The administration of L-DOPA has been shown to enhance mGlu_5_ expression in the striatum, and co-administration with MPEP in 6-OHDA-lesioned rats is able to reduce this striatal overexpression (Huang et al., 2018; Zhang et al., 2021). Similarly, the L-DOPA treatment of MPTP monkeys has been observed to increase mGlu_5_ receptor-specific binding in the basal ganglia as compared to control animals, but not in animals who had been co-administered L-DOPA and MPEP (Morin et al., 2013a). This suggests that L-DOPA-induced motor complications are associated with enhanced mGlu_5_ expression, which can be reduced using an mGlu_5_ antagonist.

The accumulation of misfolded proteins in the endoplasmic reticulum (ER) leads to ER stress in a pathway known as the unfolded protein response (UPR) which has been suggested to play a role in the pathogenesis of PD (Mercado et al., 2016). The upregulation of mGlu_5_ has been observed to induce ER stress (Gu et al., 2021). Overexpression or activation of mGlu_5_ with an agonist increased ER stress and DNA damage in primary neurons by activating ERK and JNK signaling pathways. This resulted in neuronal damage which was attenuated by pre-treatment with MPEP. These findings link the upregulation of mGlu_5_ to neurotoxicity and provide a mechanism by which mGlu_5_ antagonism may be neuroprotective in PD.

Another key process in the pathogenesis of PD is axonal degeneration (Salvadores et al., 2017). Dopaminergic neurons in the putamen are almost completely lost from 4 years after a PD diagnosis (Kordower et al., 2013). These dopaminergic neurons play as essential role in motor control and their degeneration leads to the profound motor impairments seen in PD patients (Howe and Dombeck, 2016). The downregulation of mGlu_5_ expression in 6-OHDA-lesioned primary neurons and rats treated with MPEP resulted in a reduction in axonal degeneration and attenuated 6-ODHA-activated Ca^2+^ increases (Zhang et al., 2021). In addition, this study demonstrated ERK phosphorylation and the activation of calpain play a key role in the axonal degeneration that 6-ODHA induces, both of which are also inhibited by mGlu_5_ antagonism.

Due to the therapeutic potential of mGlu_5_ antagonism in animal models of PD, several clinical trials have been carried out to investigate the efficacy of mGlu_5_ NAMs in LID PD patients. Both mavoglurant (Berg et al., 2011; clinicaltrials.gov: NCT00582673, NCT00888004; Stocchi et al., 2013; clinicaltrials.gov: NCT00986414) and dipraglurant (Tison et al., 2016; clinicaltrials.gov: NCT01336088) have been shown to have anti-dyskinetic activity in patients, without any worsening of motor symptoms. However, a meta-analysis comparing mavoglurant to placebo across randomized controlled trials found mavoglurant to be inconsistent in reducing off time in LID in PD patients, but treatment was associated with lower abnormal involuntary movements (Negida et al., 2021). To date, the only approved treatment for PD-LID is amantadine, a weak NMDA antagonist that gives increases in glutamate suggesting that in more severe PD patients that develop LID this is a more effective treatment strategy. Whether chronic mGlu_5_ NAM treatment is neuroprotective and could delay the onset LID still needs to be assessed empirically in PD.

## Neuroinflammation

A chronic neuroinflammatory response is a common pathological hallmark found across neurodegenerative diseases, including those discussed in this review. This inflammatory response is often characterized by the upregulation of neuroinflammatory cell markers, such as ionized calcium-binding adapter molecule 1 (Iba-1) and glial fibrillary acidic protein (GFAP). In addition, glial cells undergo changes in morphology and gene expression after injury or during disease (Hasel and Liddelow, 2021). These responses to pathological insult are rapid and profound, and are diverse in regards to space, time, sex and cell-subtype (Liddelow and Barres, 2017). Until recently, it was thought that this neuroinflammatory response was secondary to neuronal loss. However, there is now accumulating evidence to suggest that neurodegeneration can occur, at least in part, due to neuroinflammation (Ransohoff, 2016).

**FIGURE 2:**
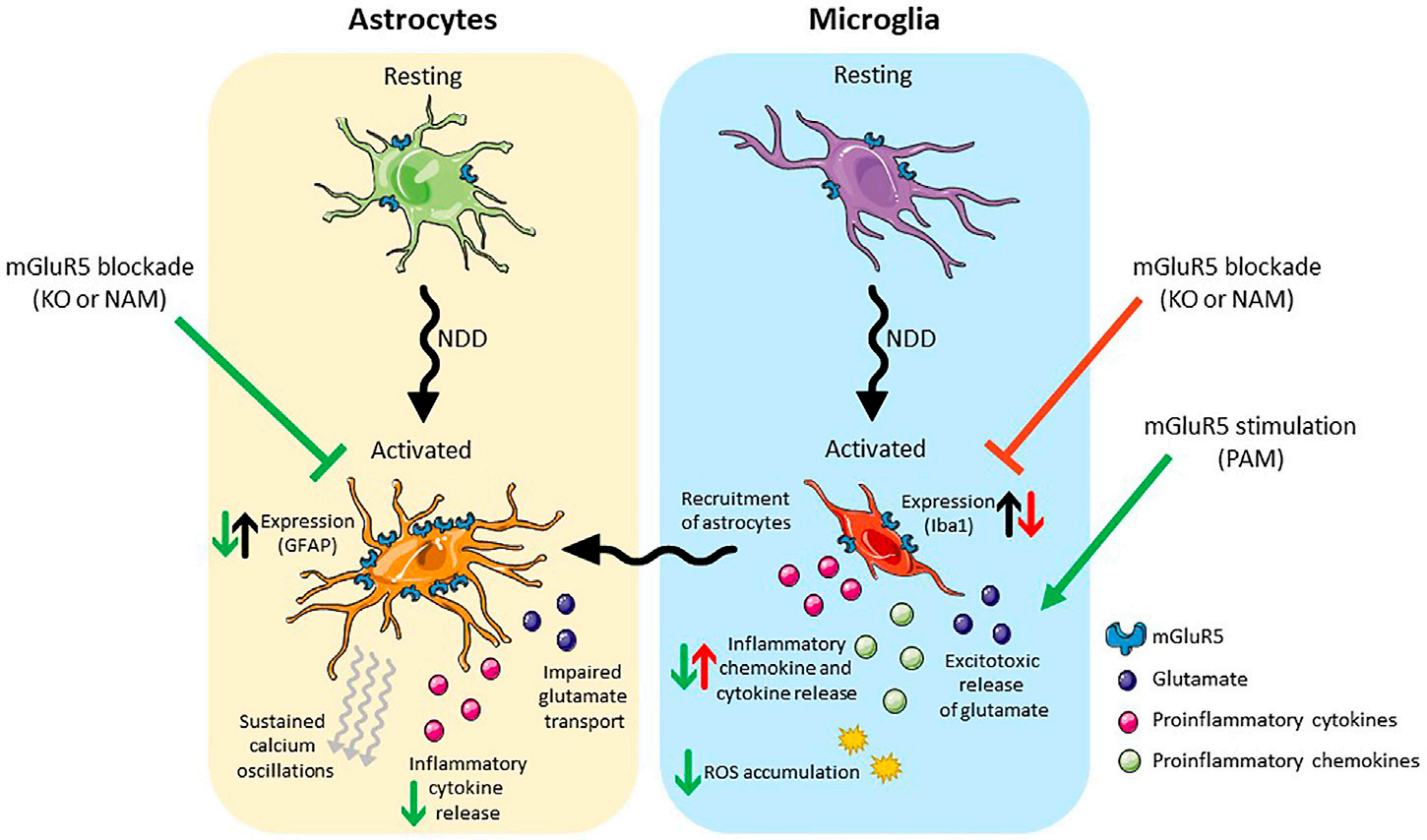
Schematic presentation of the effects of mGlu_5_ allosteric modulation on astrocytes (left) and microglia (right). Under pathological conditions both cell types take on an activated phenotype. Activated astrocytes have an increase in mGlu_5_ expression, sustained calcium oscillations, impaired glutamate transport and release pro-inflammatory cytokines. Blockade of mGlu_5_, either genetically or pharmacologically, is neuroprotective, reducing GFAP expression and the release of inflammatory cytokines. Activated microglia release inflammatory cytokines/chemokines and excitotoxic levels of glutamate and result in the ROS accumulation. In addition, they recruit activated astrocytes. Blockade of mGlu_5_, either genetically or pharmacologically reduces the expression of Iba-1 but is neurotoxic in that it results in an increase in inflammatory chemokine/cytokine release. Stimulation of mGlu_5_ with a PAM, on the other hand, is neuroprotective and reduces the release of inflammatory cytokines/chemokines and the accumulation of ROS. Abbreviations: NDD, neurodegenerative disease; KO, knockout; NAM, negative allosteric modulator; PAM, positive allosteric modulator; GFAP, Glial fibrillary acidic protein; Iba-1, Ionised calcium binding adaptor molecule 1; ROS, reactive oxygen species. Figure created using Reactome Icon Library, licensed under CC BY 4.0 (Sidiropoulos et al., 2017).

As previously mentioned, mGlu_5_ is expressed on both astrocytes and microglia. Astrocytic mGlu_5_ plays a key role in regulating excitatory transmission. Upon activation of mGlu_5_ on astrocytes, calcium oscillations are induced (Bradley et al., 2011) and glutamate is released, which subsequently induces a slow, intracellular current in neurons (Loane et al., 2012). Additionally, activation of astrocytic mGlu_5_ results in increased astrocyte proliferation (Kanumilli and Roberts, 2006), the release neurotrophic factors such as BNDF (Jean et al., 2008), and increases the activity of the glutamate transporter GLT1 leading to rapid glutamate uptake in astrocytes (Vermeiren et al., 2005). The physiological role of mGlu_5_ in microglia is less clear. This review focusses specifically on therapeutic potential of targeting of astrocytic and microglial mGlu_5_ in neurodegenerative disease (Figure 2).

### Astrocytes

In neurodegenerative diseases (as well as after infection or injury), astrocytes undergo a transition from a resting state to a reactive state (for an in-depth review see Escartin et al., 2021). The up-regulation of GFAP is a key feature of the majority of reactive astrocytes, but other markers are also upregulated such as nestin (Moreels et al., 2008), vimentin (Yamada et al., 1992), and aldolase-C (Halford et al., 2017). In addition, reactive astrocytes take on an altered morphology, with their star-like processes becoming elongated and stretching towards injury sites (Schiweck et al.,2018). Reactive astrocytes are diverse in their response to insult and take on specific states in different models of disease. It had been proposed that astrocytes took on one of two phenotypes, labelled “A1” and “A2” (Zamanian et al., 2012; Liddelow and Barres, 2017), with the former being found in chronic neuroinflammatory conditions, and the latter being found after ischemic stroke. However, the field is now moving away from these terms as it is evident that there is a large amount of heterogeneity in the responses of astrocytes to pathological conditions (Hasel and Liddelow, 2021).

Under pathophysiological conditions, mGlu_5_ is overexpressed in reactive astrocytes in AD (Shrivastava et al., 2013), ALS (Aronica et al., 2001), and epilepsy (Aronica et al., 2001). Similarly, *in vitro*, mGlu_5_ has been observed to be upregulated in cultured astrocytes after exposure to Aβ oligomers (Casley, et al., 2009), and in diseased astrocytes isolated from SOD^G93A^ rodents as compared to controls (Vermeiren et al., 2006). Upon activation, astrocytic mGlu_5_ initiates Ca^2+^ oscillations (Bradley et al., 2011). Aβ oligomers in AD and α-synuclein in PD are able to bind to and activate mGlu_5_, which could initiate sustained Ca^2+^ oscillations and lead to excitotoxicity (Price et al., 2010; Spampinato et al., 2018). Furthermore, in activated astrocytes prepared from SOD1^G93A^ mice, the overexpression of mGlu_5_ significantly impaired glutamate transport and resulted in glutamate-induced excitotoxicity (Vermeiren et al., 2006). In a similar ALS primary astrocyte culture, the typical mGlu_5_-induced Ca^2+^ oscillations showed a shift to a sustained plateau at the peak of the first oscillation (Vergouts et al., 2018). This was shown to be regulated by protein kinase C epsilon (PKCε) as increasing PKCε expression restored the mGlu_5_-induced Ca^2+^ oscillations. Moreover, astrocytes derived from ALS mice are highly vulnerable to glutamate as compared to wild-type astrocytes. This increased vulnerability is mediated *via* the activation of mGlu_5_ and results in astrocyte degeneration which is reversed by the blockade of mGlu_5_ signaling *in vivo* using MPEP (Rossi et al., 2008). Calcium signaling and glutamate transport play key roles in astrocyte-neuron communication, and their disruption may play a key role in the pathology of neurodegenerative diseases (Thibault et al., 2007; Pekny and Pekna, 2014).

The elevated expression of astrocytic markers seen in AD, HD and ALS mice is reduced with the genetic and pharmacological blockade of mGlu_5_ (Hamilton et al., 2016; Abd-Elrahman et al., 2017; Bonifacino et al., 2017; Abd-Elrahman KS. et al., 2020). In addition, pharmacological inhibition of mGlu_5_ prevents the secretion of inflammatory cytokines from astrocytes (Shah et al., 2012). Taken together, these findings suggest that the inhibition of astrocytic mGlu_5_ may lead to a reduction in the neurotoxic inflammatory state seen in neurodegenerative disease, thereby contributing to the disease-modifying effects seen in rodent models after mGlu_5_ NAM treatment.

### Microglia

Like astrocytes, microglia undergo a transformation to a reactive phenotype after pathological insult. Reactive microglia have retracted processes and an enlarged nucleus. Traditionally, reactive microglia were also characterized into two groups: “M1” and “M2”. M1 was considered a pro-inflammatory state, whereas M2 microglia were thought to be anti-inflammatory. Microglia activation is now known to be broader and more diverse (Bachiller et al., 2018). For example, a unique subtype of reactive microglia, diseased-associated microglia (DAM), has been observed in AD and ALS models, and is protective in nature (Keren-Shaul et al., 2017).

As with astrogliosis, the genetic or pharmacological blockade of mGlu_5_ in AD and ALS rodent models led to a reduction in microgliosis (Bonifacino et al., 2017; Abd-Elrahman K. S. et al., 2020; Milanese et al., 2021). As reactive microglia have a neurotoxic effect mediated by the secretion of proinflammatory cytokines and chemokines and the release of excitotoxic levels of glutamate (Barger and Basile, 2001; Hemonnot et al., 2019), it could be assumed that reducing their levels would be neuroprotective. To the contrary, the inhibition of mGlu_5_ in microglia drives them towards a pro-inflammatory state (Chantong et al., 2014). In cultured microglia activated by the overexpression of α-synuclein, the addition of an mGlu_5_ NAM enhanced inflammation in these cells by exacerbating inflammatory signaling pathways (Zhang et al., 2021).

There is mounting evidence to suggest that activation of microglial mGlu_5_ could be important for counteracting the neurotoxic effect of microglia in neurodegenerative disease. Activation of mGlu_5_ with an agonist or PAM has been shown to consistently reduce microglial activation and the associated inflammation in primary microglial cultures and cells lines challenged with pro-inflammatory molecules, including Aβ and α-synuclein (Byrnes et al., 2009; Farso, O’Shea and Beart, 2009; Loane et al., 2009, 2013, 2014; Piers, Heales and Pocock, 2011). This effect was not observed in primary cultures from mGlu_5_ knockout mice nor in the presence of an mGlu_5_ antagonist. Moreover, activating microglial mGlu_5_ reduces apoptosis, reduced reactive oxygen species accumulation, and increases the production of brain-derived neurotrophic factor (Ye et al., 2017). *In vivo*, mGlu_5_ PAM administration reduces neuronal loss in a mouse model of traumatic brain injury by reducing the inflammation induced by microglia (Loane et al., 2014). Whilst it is clear that mGlu_5_ signaling in astrocytes can contribute to neurotoxicity, these data suggest that mGlu_5_ signaling in microglia may be neuroprotective.

It is important to consider the differing effects of mGlu_5_ agonists, antagonists, NAMs and PAMs on glial cells when developing ligands for the treatment of neurodegeneration. In addition, the activation of microglia in disease results in the recruitment of astrocytes and induces their conversion into a reactive phenotype (Liddelow and Barres, 2017). Thus, the benefit of activating microglia in disease with an mGlu_5_ PAM may be limited by the subsequent neurotoxic response from astrocytes. Similarly, a NAM that is neuroprotective in pre-clinical and clinical models may have a detrimental effect by driving a proinflammatory microglial response, even though it may reduce astrocyte-induced excitotoxicity. As targeting neuroinflammatory processes may be of benefit across multiple neurodegenerative diseases and targeting mGlu_5_ modulates inflammation, it will be important to better understand the relative contribution of each cell type to pathology in order to select the most effective ligands to treat disease. It is not yet clear whether mGlu_5_ will need to be activated or inhibited in order to most effectively modulate disease pathology and progression via its effect on neuroinflammation.

## Conclusion

Much of the research into the role of mGlu_5_ receptors in neurodegenerative diseases has focused on the use of specific allosteric modulators, both positive and negative. The neuroprotective effects of both the up and downregulation of mGlu_5_ receptor signaling emphasise the role mGlu_5_ plays in the pathology of these diseases and highlights its potential as a therapeutic target. In Alzheimer’s disease, the genetic deletion or pharmacological blockade of mGlu_5_ improved cognition and reduced disease-related pathology in rodent models of disease. These benefits were paralleled by an increase in autophagy and a reduction in neuroinflammation. However, the genetic deletion of mGlu_5_ in healthy mice worsens learning and memory processes and accelerates neurodegeneration. Enhancing mGlu_5_ signaling with mGlu_5_ PAMs has also been beneficial in rodent models of AD, preventing neuronal loss but not improving cognition. Similarly, both NAMs and PAMs have been neuroprotective in rodent models of Huntington’s disease. However, PAMs may have a potential excitotoxic effect. In ALS and PD, it is mGlu_5_ NAMs that have been associated with improved disease pathology and cognition. However, no drug has yet received regulatory approval for these indications.

It is evident that glial mGlu_5_, specifically mGlu_5_ receptors expressed on astrocytes and microglia, plays a role in neurodegeneration. Astrocytes are upregulated in the brains of patients and rodent models and mGlu_5_ activation contributes to the neurotoxic effect they exert. Microglial activation, on the other hand, is neuroprotective against inflammation. Fundamental questions remain surrounding the opposing role of mGlu_5_ in astrocytes and microglia and the precise contribution of mGlu_5_ signaling in these cells to inflammation.

In conclusion, the neuroprotective effect of modulating mGlu_5_ signaling highlights this receptor as a promising therapeutic target for the treatment of neurodegenerative diseases. Furthermore, its expression on glial cells and ability to modulate inflammation suggests that it play a neuroprotective role by resolving common pathologies across diseases. Ongoing clinical trials using mGlu_5_ modulators in neurodegenerative diseases, including AD and PD, as well as other neurological conditions, such as FXS, will help elucidate the role of the mGlu_5_ in different disease mechanisms and its therapeutic potential in neurodegenerative disorders. The outcomes of these studies will have a major impact on completing the picture of the role of mGlu_5_ in neurodegeneration and its potential as a drug target for the treatment of neurodegenerative diseases.
